# Comparison Between Antenatal and Postnatal Colostrum From Women With and Without Type 1 Diabetes

**DOI:** 10.1177/08903344251318285

**Published:** 2025-03-12

**Authors:** Alexandra Goldberg, Hans Pettersson, Cecilia Ekéus, Carina Ursing, Eva Wiberg-Itzel, Joanna Tingström

**Affiliations:** 1 Department of Clinical Science and Education, Karolinska Institutet Soder Hospital, Stockholm, Sweden; 2 Department of Women’s and Children’s Health, Uppsala University, Uppsala, Sweden

**Keywords:** antenatal breastmilk expression, antenatal colostrum, antenatal colostrum expression, breastfeeding, breastfeeding support, colostrum, diabetes, exclusive breastfeeding, formula feeding, macronutrition, Type 1 Diabetes Mellitus

## Abstract

**Background:**

Supplementary feeding, colostrum or, in some countries, commercial milk formula, is given to newborns of women with Type 1 diabetes to prevent neonatal hypoglycemia. Few studies have explored the content of colostrum from women with Type 1 diabetes.

**Research Aims:**

This study aimed to investigate the macronutrients in colostrum collected during pregnancy and in the early postpartum period to compare colostrum contents in women with and without Type 1 diabetes.

**Methods:**

In this cohort study, we collected colostrum among 20 women, 10 with and 10 without Type 1 diabetes, at 10 different time points in gestational weeks 36–40 and postpartum Days 1–5. We measured carbohydrates, protein, fat, and kilocalories in colostrum using a human milk analyzer; and we analyzed data using linear mixed models. In a follow-up analysis, we compared the content of colostrum from Day 1 with the nutritional values provided on the commercial milk formula, using a one-sample *t* test.

**Results:**

There were no mean differences in carbohydrates (6.6 g/100 ml; 95% CI [6.3, 6.9] vs. 6.7 g/100 ml; 95% CI [6.4, 7.0] *p* = 0.29); kilocalories (71.1 kcal/100 ml; 95% CI [62.9, 79.3] vs. 85.3 kcal/100 ml; 95% CI [77.2, 93.3] *p* = 0.21], and fat (2.7 g/100 ml; 95% CI [1.8, 3.6] vs. 2.3 g/100 ml; 95% CI [1.4, 3.2] *p* = 0.55) in colostrum when comparing women with and without Type 1 diabetes. However, antenatal protein differed at all timepoints tested (*p* = 0.01). Colostrum macronutrients on Day 1 differed from that of commercial milk formula and all other colostrum time points, except Gestational Week 38.

**Conclusions:**

Our study provides insights into antenatal and postnatal colostrum macronutrients among women with and without Type 1 diabetes. Further studies are needed to understand the effects of supplementary feeding using antenatal or postnatal colostrum or commercial milk formula on neonatal hypoglycemia.

## Key Messages

- Antenatal colostrum differs from postnatal colostrum and between women with and without Type 1 diabetes. The differences need to be explored to better evaluate if the macronutrient in antenatal colostrum aligns with the nutritional needs of the newborns of mothers with Type 1 diabetes.
- The researchers found no significant differences in colostrum between women with and without Type 1 diabetes, except protein at various antenatal time points.
- The contents of antenatal colostrum from both women with and without Type 1 diabetes indicate it is a better supplement for newborns compared to commercial milk formula.
- More research is needed on the implications of protein content in antenatal colostrum.

## Background

Newborns of mothers with Type 1 diabetes (T1D)—that is, total insulin deficiency—are at a higher risk of developing neonatal hypoglycemia compared with the general newborn population (American Diabetes Association, 2020; Thinesh Kumar et al., 2018). To avoid hypoglycemia, supplementary feeding after breastfeeding—here defined as “the child has received breastmilk (direct from the breast or expressed)” (World Health Organization [WHO], 1991), is recommended for this group in some countries. In Sweden, only commercial milk formula was used for supplementary feeding until 2019 (Wackernagel et al., 2020), when antenatal colostrum (AC) was introduced as an option for supplementary feeding. AC can be obtained by antenatal colostrum expression (ACE). ACE from gestational week (GW) 36 does not cause any harm, such as provoking premature birth. If ACE is performed by first-time mothers with diabetes, their newborns are more often breastfed exclusively during the first 24 hours after birth (Forster et al., 2017). The American Academy of Pediatrics recommends that birth hospitals implement practices that increase breastfeeding exclusivity (Meek et al., 2022). According to the World Health Organization (WHO, 2015), exclusive breastfeeding is defined as only human milk (HM) and no other fluids, such as water. Commercial milk formula intake is associated with decreased breastfeeding and can elevate the newborn’s insulin production, leading to an increased risk of hypoglycemia (Dalsgaard et al., 2019; McCoy & Heggie, 2020; Slupsky et al., 2017).

However, as an option instead of a commercial milk formula, ACE is only recommended in some regions of Sweden. Therefore, most newborns of women with T1D in Sweden receive supplementary feeding with commercial milk formula, regardless of whether they have been breastfed first or received less than 10–20 ml of colostrum after breastfeeding (Wackernagel et al., 2020). In those regions that recommend ACE, it has yet to become a common practice for most women with T1D. The lack of scientific evidence on the difference in the content between commercial milk formula and AC and postnatal colostrum (PC) from day one is the reason Swedish guidelines do not distinguish between colostrum and commercial milk formula in terms of the quantities when supplementary feeding is recommended for newborns at risk of hypoglycemia. As far as we know, only one study has analyzed AC, and only during GW 37 in women without T1D (Rosenberg et al., 2021).

Furthermore, there is a vast difference in the contents of PCs among women. Specific differences in kilocalories (kcal), carbohydrates, and fat among women’s PCs have been described (Ballard & Morrow, 2013). The question as to whether PC also differs between women with and without T1D still needs to be clarified. As far as we know, no study has been conducted on carbohydrates in AC or PC in women with T1D (Rassie et al, 2021). Moreover, few studies have explored the differences between commercial milk formula and PC from Day 1, and no study has compared commercial milk formula with AC (Kim & Yi, 2020).

Additionally, the content of AC from women with T1D needs to be explored to make recommendations on how or if AC can be used as supplementary feeding. Specifically, AC from women with T1D needs to be compared with both their PC and with AC and PC from women without T1D, to evaluate better whether the macronutrient content aligns with a newborn’s nutritional needs. This study aimed to investigate the macronutrients in colostrum collected during pregnancy and in the early postpartum period, and to compare colostrum contents in women with and without Type 1 diabetes.

## Methods

### Research Design

In this cohort study, we collected colostrum from 20 women (10 women with Type 1 diabetes and 10 without Type 1 diabetes) during GW 36–40 and postpartum Days 1 to 5. A cohort study was found to be sufficient for considering the macronutrient contents of colostrum, in terms of carbohydrates, protein, fat, and kcal, which were compared at 10 time points with Type 1 diabetes as the exposure. To provide the best recommendations on what supplemental feeding to provide to newborns of mothers with T1D, more research needed to understand the differences between PC and AC on Day 1 and PC and commercial milk formula on Days 2–5. Therefore, we performed a follow-up analysis. We compared the macronutrient contents of PC on Day 1 with all the other colostrum time points and with the nutritional values provided for commercial milk formula.

The ethical board approved this study on February 16, 2021 (Dnr: 2020-06042).

### Setting and Relevant Context

All participants of this study were native Swedish individuals and were living with a partner or married. In 2021, 60.9% of Swedish women breastfed exclusively at 2 months and 11.9% did so at 6 months (Socialstyrelsen, 2023). The Swedish Food Agency (2022) agrees with the WHO (2019) recommendation of exclusive breastfeeding for the first 6 months and continued breastfeeding for 2 years or beyond. Uninterrupted skin-to-skin for the first 1–2 hours after birth is standard care in Sweden to encourage initial breastfeeding. Up to 40% of full-term newborns whose mothers do not have a diagnosis of diabetes during pregnancy are fed with commercial milk formula after birth during the hospital stay (Skogsdal et al., 2022).

### Sample

The target sample for this study was pregnant women with and without T1D who intended to breastfeed for any duration of time (Labbok & Starling, 2012) and who were living in Sweden. The standard care in antenatal clinics in Sweden includes the midwife who provides breastfeeding information around GW 32. Pregnant women with T1D before pregnancy were recruited for inclusion at their specialist antenatal clinic by their midwife after receiving breastfeeding information if they expressed the desire to breastfeed, while women without T1D were recruited at four antenatal clinics by their midwives after receiving breastfeeding information if they intended to breastfeed (Labbok & Starling, 2012). Information was also distributed by posters at antenatal clinics. Women without T1D were excluded if they had any form of diabetes. The participants did not receive any compensation for participating in the study.

Twenty participants were included (*N* = 20; with T1D *n* = 10, without T1D *n* = 10). Recruitment of participants ended after colostrum samples were collected from 10 women with T1D and 10 without T1D. A small sample size might not be adequately powered to detect a difference between the groups, with a high risk of Type II error. No formal power analysis was done, considering that there is only one previous study on AC. However, according to Rassie et al. (2021), fewer than 10 participants were included in previous comparable studies on PC. Twenty participants was therefore considered an appropriate sample for this study. Follow-up with the women was accomplished via text messages and phone calls from one of the researchers (AG).

### Measurements

Data on age, education, body mass index (BMI), parity, hypertension, pregnancy-induced hypertension, preeclampsia, and information about the birth were collected from participants’ medical records. When collecting the data, the researcher officially established their T1D status by obtaining the T1D diagnosis from the participant's medical records. Data on hemoglobin A1c (HbA1c) for each trimester and plasma glucose (p-glucose) during birth were also recorded.

Each newborn’s gestational age (GA) and birthweight were recorded and categorized as appropriate, large, or small for GA. The categorization was based on the mean for average fetal growth weights (≤ 2 I above and ≤ 2 *SD* below), (> 2 *SD* above), or (> 2 *SD* below), respectively, according to Swedish reference data (Marsal et al., 1996).

In the colostrum, carbohydrates, true protein (protein), fat (g/100 ml), and kcal (kcal/100 ml) were measured with the Human Milk Analyzer (Miris, Uppsala, Sweden). The colostrum samples were brought in a cooler, heated, and then homogenized with an ultrasonic technique (MIRIS milk sonicator, Miris, Uppsala, Sweden) before analysis.

### Data Collection

The data from the participants’ medical records were collected between 2021 and 2023 by one of the researchers (AG), kept confidential, and managed according to “good clinical practice” and the General Data Protection Regulation. The data were safely stored, and only one of the researchers (AG) had access to the information. All participants received written and oral information about the study and could end their participation without affecting their care. All participants signed a written form of consent.

The women with T1D received ACE instructions orally and in writing from their midwife at the specialist antenatal care. The women without T1D received written instructions for ACE by post, and one of the researchers (AG) explained colostrum expression by hand orally over the telephone. All the women received medicine cups with lids for collecting the colostrum.

ACE was performed weekly from GW 36 + 0 until the participants gave birth. The standard care for women with T1D at this specialist antenatal clinic is to encourage them to perform ACE twice a day at their chosen time and reserve some of the colostrum for the study and some for the newborn. The participants without T1D were encouraged to express colostrum in the morning, but the sample was still considered valid if they expressed colostrum later in the day.

Postpartum ACE was performed manually once a day for the first 5 postpartum days. All the participants were encouraged to express colostrum in the morning, although none of the samples were excluded if the colostrum was expressed later in the day. The time when the colostrum was expressed was not noted for all samples.

The participants stored the colostrum in their freezers at home and in the postnatal ward freezer during their hospital stay. Samples (1–10 ml) were collected, and the amounts differed widely from woman to woman. All participants were encouraged to collect even small drops and up to 10 ml. Hence, the amount that was available for analysis was not known beforehand. The person who analyzed the study material at the Miris laboratory was not involved in any other part of the study.

Commercial milk formula was not measured in the same way as colostrum. The nutritional value of the commercial milk formula was recorded based on the nutritional label of the most commonly used commercial milk formula in Swedish hospitals (Baby Semp 1, Semper^®^).

### Data Analysis

To compare the participants with and without T1D, we used the independent *t* test for the means, with normally distributed continuous variables, and the Mann–Whitney *U* test for the variables with a skewed distribution. Normality was assessed by the Shapiro-Wilk test. To compare proportions the chi-squared test, or when the sample sizes were small, Fisher’s exact test was applied.

We used linear mixed models to investigate whether there were any differences among the time points or between the groups (participants with and without T1D) across all 10 time points. This type of model is the preferred choice for repeated measures with unequal time points and partially missing data (Järnbert-Pettersson & Vixner, 2018). We separately analyzed each outcome for the carbohydrates, fat, kcal, and protein. The model strategy involved the following steps: First, in a fixed model with an intercept and group and time as variables, covariance structures for a first-order autoregressive model—AR(1)—and Unstructured were compared for each outcome. The AR(1) displayed a better covariance structure for all variables, according to the -2 log-likelihood test. Second, the interaction between time and group was tested to examine whether the outcome difference between the groups (women with and without T1D) differed between the time points. Third, if overall differences were found regarding time, pairwise comparisons between PC on day one and at the other time points were made.

The means of all participants’ carbohydrates, fat, kcal, and protein in their PC from Day 1 was compared with the nutritional values provided on commercial milk formula using the one-sample *t* test. A *p* value of < 0.05 was considered statistically significant. All presented *p* values were two-sided. All analyses were performed using SPSS (Version 28).

## Results

The study included 20 participants, *n* = 10 (50%) with T1D and *n* = 10 (50%) without T1D. The women with T1D had a higher BMI and gave birth at a lower GA compared to those without T1D. None of the newborns developed hypoglycemia. The women without T1D had a spontaneous onset of birth more often and were more often breastfeeding exclusively from birth to Day 5 in comparison to the women with T1D. None of the newborns lost 10% or more of their birth weight (Tables 1 and 2). In total, 160 colostrum samples were analyzed and a statistically significant difference between the colostrum timepoints was found.

**Table 1.** Characteristics of the Study Sample for Participants With T1D and Without T1D (*N* = 20).

	T1D (*n* = 10)	No T1D (*n* = 10)		
Characteristics	*n* (%)	*n* (%)	***χ*** ^2^	*p* ^a^
Primipara	6 (60)	3 (30)		0.37
Smoking	0 (0)	1 (10)		1.00
University degree	8 (80)	10 (10)		0.47
Hypothyroidism	2 (20)	2 (20)		1.00
PE	2 (20)	0 (0)		0.47
Birth starts with contractions	2 (20)	8 (80)	7.200	0.02
Epidural	7 (70)	3 (30)	3.200	0.18
Oxytocin Augmentation	6 (60)	4 (40)	0.800	0.66
Partus Normalis	7 (70)	9 (90)		0.58
Vacuum Extraction	2 (20)	1 (10)		1.00
Elective cesarean section	1 (10)	0 (0)	0.50	1.00
Newborns of participants:				
LGA	2 (20)	1 (10)		1.00
SGA	0 (0)	0 (0)		
Female	6 (60)	4 (40)	0.800	0.66
Hypoglycemia in newborn	0 (0)	0 (0)^a^		
Weight loss ≥ 10%	0 (0)	0 (0)		
Exclusive breastfeeding from birth until day five	3 (30)	9 (90)		0.02
Exclusive breastfeeding at first checkup	6 (60)	10 (100)		0.09
Exclusive breastfeeding at 2 months	10 (100)	8 (80)		0.47
Only breastfeeding at 4 months^b^	9 (90)	9 (90)		1.00
Only breastfeeding at 6 months^b^	2 (20)	7 (70)		0.07

*Note.* T1D = Type 1 diabetes. PE = preeclampsia. LGA = large for gestational age. SGA = small for gestational age. *P* values calculated with Fischer Exact (without a test statistic) or ***χ***^2^ (with a test statistic). *P* value < 0.05 was considered statistically significant.

a Only one newborn was tested in the healthy group. ^b^No commercial milk formula and taste sensations with food according to standard recommendations in Sweden.

**Table 2.** Characteristics of the Study Sample for Participants With T1D and Without T1D (*N* = 20).

	T1D (*n* = 10)	No T1D (*n* = 10)		
Characteristics	*M* (*SD*)	*M* (*SD*)	*t*	*p*
Age (years)	32 (5)	32 (5)	−0.32	0.71
BMI (kg/m) at first antenatal enrollment	26.7 (3.4)	21.2 (2.1)	4.25	<0.001
Newborns of participants				
Gestational age (weeks + days)	39 + 0 (8)	40 + 1 (4)	−2.75	0.01
Weight (g)	3607 (510)	3598 (478)	−0.4	0.98

*Note.* T1D = Type 1 diabetes, BMI = body mass index, *M* and *SD* are used to represent mean and standard deviation, respectively. *P* value < 0.05 was considered statistically significant.

**Figure 1.**
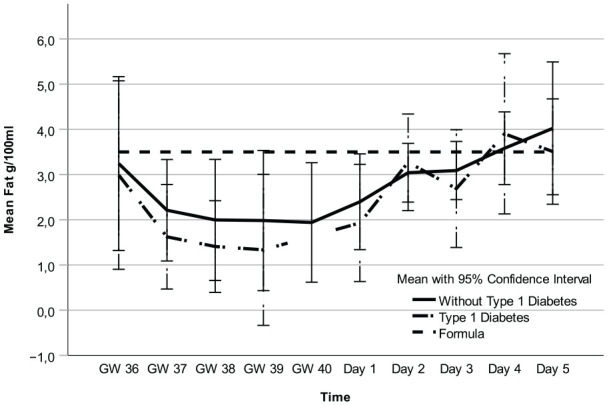
Fat in Colostrum and Formula Over Time. *Note.* Gestational Weeks (GW) 36–40 and Postpartum Days 1–5.

**Figure 2.**
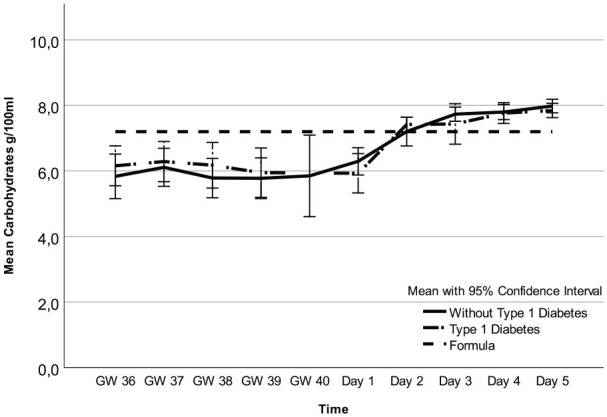
Carbohydrates in Colostrum and Formula Over Time. *Note.* Gestational Weeks (GW) 36–40 and Postpartum Days 1–5.

**Figure 3.**
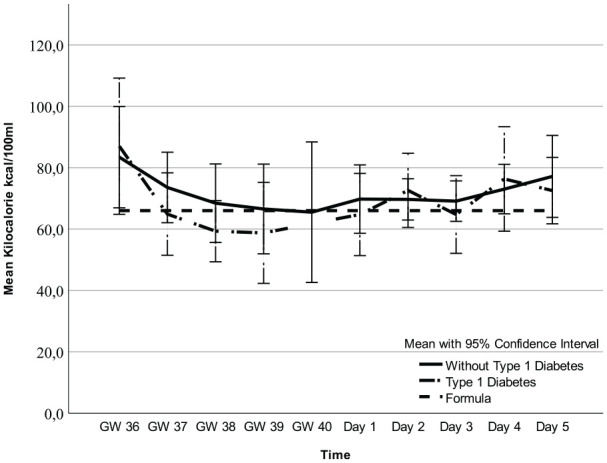
Kilocalorie in Colostrum and Formula Over Time. *Note.* Gestational Weeks (GW) 36–40 and Postpartum Days 1–5.

**Figure 4.**
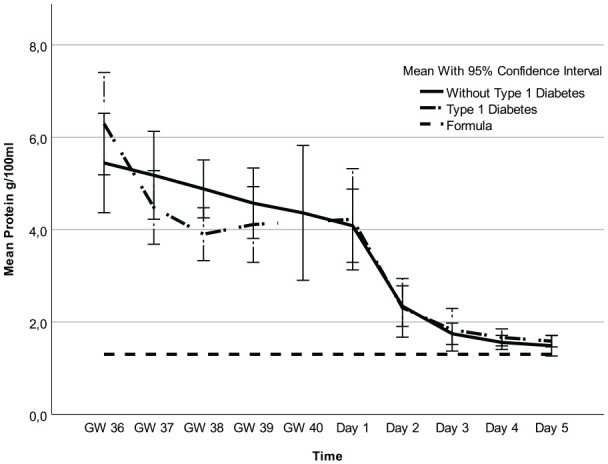
Protein in Colostrum and Formula Over Time. *Note.* Gestational Weeks (GW) 36–40 and Postpartum Days 1–5.

Regarding fat, carbohydrates, and kcal, there were no mean differences between participants with and without T1D (Figures 1–3, Table 3 and Supplemental Table 1a–c, which can be found in the online supplemental material) and no interactions between these two groups of women and time points (2.7 g/100 ml; 95% CI [1.8, 3.6] vs. 2.3 g/100 ml; 95% CI [1.4, 3.2] *p* = 0.55), 6.6 g/100 ml; 95% CI [6.3, 6.9] vs. 6.7 g/100 ml; 95% CI [6.4, 7.0] *p* = 0.29), and 71.1 kcal/100 ml; 95% CI [62.9, 79.3] vs. 85.3 kcal/100 ml; 95% CI [77.2, 93.3]; *p* = 0.21, respectively). However, there was an interaction for protein (*p* = 0.01), implying that the difference in colostrum between women with and without T1D fluctuated during some time points (Figure 4, Table 3, and Supplemental Table 1d). Finally, a random intercept was tested for each outcome, and none were significant. The missing values of colostrum samples for each time point are presented in the online Supplemental Material.

**Table 3.** Test of Macronutrients in Colostrum Between all Timepoints (GWs 36–40 and Postpartum Days 1–5) and Between Participants With and Without T1D (*N* = 20).

	Comparisons		
	Participants With and Without TID	All Timepoints^a^	Participants With and Without T1D Between all Timepoints^b^
Macronutrients	*p*	*p*	*p*
Fat	0.50	< 0.001	0.55
Carbohydrates	0.77	< 0.001	0.29
Protein	0.75	< 0.001	0.01
Kcal	0.52	< 0.001	0.21

*Note*. GW = gestational week, T1D = Type 1 diabetes, Kcal = kilocalorie, *P* value < 0.05 was considered statistically significant.

a Timepoints include GW 36, 37, 38, 39, 40 and Day of Life 1, 2, 3, 4, and 5. ^b^Interaction between time and group. Estimated fixed coefficients and 95% CI from the mixed models for each outcome can be found online in Supplemental Table 1a–d.

Analysis of all colostrum samples indicated differences in fat, carbohydrates, kcal, and protein across all time points (Figures 1–4 and Table 3). Protein was the macronutrient that had the most time points with significant differences compared to PC on Day 1 (Table 4). AC and PC on Day 1 contained higher protein levels than PC on Days 2–5 (Figure 3 and Table 4). Fat and carbohydrate contents of AC, together with PC on Day 1, were lower than on all the other PC days (Figures 1 and 2 and Table 4). Kcal levels stayed the same across all time points except for the higher levels in GW 36 (66.8 kcal/100 ml 95% CI [58.7, 75.0] vs. 85.3 kcal/100 ml; 95% CI [77.2, 93.3] *p* < 0.001; Figure 4 and Table 4). A notable disparity was revealed between commercial milk formula and PC on Day 1. We observed that commercial milk formula had higher levels of fat compared to PC on day 1 (3.5 g/100 ml vs. 2.1 g/100 ml; 95% CI [1.3, –3.0], *p* = 0.002 and carbohydrates (7.2 g/100 ml vs. 6.2 g/100 ml; 95% CI [5.9, –6.5], p < 0.001, while its protein content was lower (1.3 g/100 ml vs. 4.1 g/100 ml; 95% CI [3.7, –4.6], *p* < 0.001, as shown in Figures 1–4 and Table 4.

**Table 4.** Pairwise Comparison of Mean Macronutrient Levels on Day 1 Versus Later Timepoints and Commercial Milk Formula in Participants With and Without T1D (*N* = 160 Samples Among 20 Participants With and Without T1D).

Macronutrient	Fat		Carbohydrates		Protein		Kcal	
Day	Mean (CI)	*p*	Mean (CI)	*p*	Mean (CI)	*p*	Mean (CI)	*p*
Day 1	2.1 (1.3 - 3.0)		6.2 (5.9 - 6.5)		4.1 (3.7 - 4.6)		66.8 (58.7 - 75.0)	
GW 36	3.1 (2.3 - 3.9)	0.04	6.1 (5.8 - 6.4)	0.55	5.9 (5.5 - 6.3)	< 0.001	85.3 (77.2 - 93.3)	< 0.001
GW 37	1.9 (1.1 - 2.7)	0.64	6.2 (5.9 - 6.5)	0.66	4.9 (4.5 - 5.3)	0.006	70.0 (61.9 - 77.8)	0.50
GW 38	1.6 (0.8 - 2.5)	0.23	6.0 (5.7 - 6.3)	0.22	4.4 (3.9 - 4.8)	0.40	63.4 (55.3 - 71.6)	0.43
GW 39	1.5 (0.7 - 2.4)	0.12	5.9 (5.5 - 6.2)	0.04	4.3 (3.8 - 4.8)	0.52	61.5 (53.0 - 70.0)	0.18
GW 40	1.6 (0.5 - 2.7)	0.22	5.8 (5.4 - 6.3)	0.09	5.1 (4.3 - 5.8)	0.01	65.6 (54.6 - 76.7)	0.81
Day 2	2.9 (2.1 - 3.8)	0.003	7.2 (6.9 - 7.5)	< 0.001	2.4 (1.9 - 2.8)	< 0.001	68.8 (60.5 - 77.1)	0.49
Day 3	2.9 (2.1 - 3.8)	0.02	7.6 (7.3 - 7.9)	< 0.001	1.8 (1.4 - 2.2)	< 0.001	67.3 (59.2 - 75.3)	0.90
Day 4	3.8 (2.9 - 4.6)	< 0.001	7.8 (7.5 - 8.1)	< 0.001	1.6 (1.2 - 2.0)	< 0.001	74.8 (66.8 - 82.8)	0.58
Day 5	3.7 (2.9 - 4.6)	< 0.001	7.9 (7.6 - 8.2)	< 0.001	1.5 (1.0 - 2.0)	< 0.001	74.6 (66.6 - 82.7)	0.88
Formula	3.5	0.002	7.2	< 0.001	1.3	< 0.001	66.0	0.68

*Note.* T1D = Type 1 Diabetes, GW = gestational week, Kcal = kilocalorie, *P* value < 0.05 was considered statistically significant. Fat, Carbohydrates and Protein g/100ml. Kcal/100ml.

## Discussion

As far as we know, this is the first study that analyzed AC from women with T1D and AC from GW 36 and 38–40 from women without DM1. Rosenberg et al. (2021) analyzed AC in GW 37 from women without T1D and compared it with PC on Day 3. They found AC fat and carbohydrate distribution lower, and protein distribution higher, than those of PC on Day 3. When we compared the same time points (AC in GW 37 vs. PC on Day 3), our findings confirmed theirs (Rosenberg et al., 2021). This study compared AC in GWs 36–40 with PC on Day 1. Hence, mothers with T1D are advised to provide supplementary feeding to their newborns from Day 1, and they can use AC from GWs 36–40 (Wackernagel et al., 2020).

The macronutrient patterns of AC and PC on Day 1 tend to be more similar compared to PC on Days 2–5. The composition changes during PC Days 2–5 align with the description of macronutrient changes during the onset of mature HM (Lactogenesis II, copious milk production) (Neville et al., 1991). The expelling of the placenta, followed by hormone changes, causes the alteration of PC to HM (Neville et al., 2001). Therefore, the macronutrient content on PC Day 1 is more in line with AC than with PC Days 2–5. Specifically, because the alteration starts on PC Day 1, it significantly differs from AC.

Moreover, AC and PC macronutrient carbohydrates, fat, and kcal did not differ between women with and without T1D, and normoglycemia in the third trimester could be the reason for this. Specifically, the third-trimester p-glucose would mainly impact all macronutrients except AC protein (Table 5). Evidence for the impact of p-glucose on macronutrient content, especially fat, has been strengthened by earlier research. Morceli et al. (2011) found that women with T1D and an elevated HbA1c during pregnancy had a lower colostrum fat composition than those without T1D. Van Beusekom et al. (1993) described that women with T1D and a normal HbA1c did not differ in colostrum fat composition compared to women without T1D.

**Table 5.** Hemoglobin A1C < 42 mmol/mol During Pregnancy and Plasma Glucose During Birth in Participants With Type 1 Diabetes (*n* = 10).

Timepoints	*n* ( %)
First Trimester	6 (60)
Second Trimester	10 (10)
Third Trimester	8 (80)
All three trimesters	5 (50)
Birth (plasma glucose)	
4–7 mmol/l	3 (33)
> 7 mmol/l	3 (33)

*Note*: One participant did not have the data for birth plasma glucose. Three participants had plasma glucose levels between 4–7 mmol/l during birth, and plasma glucose levels > 7 mmol/l were found in three other participants.

Furthermore, hyperglycemia in the second trimester might explain why we found a significant difference in AC protein between women with and without T1D. Hyperglycemia is thought to influence protein levels through a suboptimal insulin dynamic, as described in an animal study (Neville et al., 2013). Specifically, 90% of the women with T1D had an HbA1c > 42 mmol/mol in the second trimester (Table 5).

Protein in PC did not differ between women with and without T1D, which could be explained by the fact that most of the women with T1D achieved normoglycemia in the third trimester (Table 5). Hyperglycemia during pregnancy would then have a delayed impact on the protein levels. Earlier research is contradictory regarding evidence for a difference in protein content in PC and HM between women with and without T1D (Bitman et al., 1989; Jackson et al., 1994; Morceli et al., 2011). The small number of participants in our study could also explain the results. Further research is needed to understand the impact of p-glucose on protein levels in both AC and PC from women with T1D.

Even though the protein composition differed in AC between women with and without T1D, AC should be the primary choice when supplementary feeding is recommended to prevent hypoglycemia, if it is not possible to obtain fresh colostrum. In our follow-up analysis, the protein levels for all the women’s AC were higher than that of the formula. Formula’s significant difference in lower protein and higher carbohydrates and fat compared to PC Day 1 is well known and explained by the fact that commercial milk formula has been developed to mimic mature HM, not colostrum (Kim & Yi, 2020). Protein is essential for preventing hypoglycemia and achieving a stable p-glucose in children and adults (Spiller et al., 1987). We have not found any studies where the researchers have analyzed whether protein is an essential factor for preventing hypoglycemia in newborns at birth. Still, protein levels are critical for newborns’ growth and development (Kim & Yi, 2020).

In contrast to commercial milk formula, AC has the same or a higher protein level than PC on Day 1. It would be preferable for newborns to receive the expected or higher-than-expected quantity of protein versus a lower amount, as this is what newborns are expected to receive. The question of how the lower protein level in commercial milk formula, compared to colostrum, affects newborns’ p-glucose levels needs to be studied further.

In a retrospective cohort study by Cordero et al. (2018), newborns of women with T1D who were first fed with colostrum rather than commercial milk formula were less prone to develop hypoglycemia. Our findings relating to colostrum macronutrients may be one plausible explanation for adding support to colostrum feeding, instead of commercial milk formula, of newborns in need of supplementary feeding.

## Limitations

This study included a small number of participants, and colostrum samples from some time points were not collected from all participants. A cohort study could benefit from a larger sample size and a longer duration. Although we compared two groups and could not extend the timeframe for colostrum, we still found the cohort study suitable to answer our research question. Furthermore, all the samples were not collected at the same time during the day. Moreover, the study did not control for maternal diet, health conditions, and medication. There is also a risk of selection bias, considering that the women were recruited only in Stockholm, and those with T1D were recruited at only one clinic. Out of the 20 participants, 18 had a university degree. A high socioeconomic status is associated with a healthier diet, and diet is known to influence the content of HM (Moraeus et al., 2020; Zhang et al., 2021). These factors might contribute to the findings being less generalizable.

## Conclusions

We found that the macronutrients of AC differed from PC among women with and without T1D. The only difference in colostrum between women with and without T1D was found in AC protein. AC was more aligned with the nutritional needs of a newborn when compared to commercial milk formula. Further studies should investigate the effects of supplementary feeding using AC, PC from Day 1, and commercial milk formula, on neonatal hypoglycemia.

## Supplemental Material

sj-docx-1-jhl-10.1177_08903344251318285 – Supplemental material for Comparison Between Antenatal and Postnatal Colostrum From Women With and Without Type 1 Diabetes Supplemental material, sj-docx-1-jhl-10.1177_08903344251318285 for Comparison Between Antenatal and Postnatal Colostrum From Women With and Without Type 1 Diabetes by Alexandra Goldberg, Hans Pettersson, Cecilia Ekéus, Carina Ursing, Eva Wiberg-Itzel and Joanna Tingström in Journal of Human Lactation

sj-docx-2-jhl-10.1177_08903344251318285 – Supplemental material for Comparison Between Antenatal and Postnatal Colostrum From Women With and Without Type 1 Diabetes Supplemental material, sj-docx-2-jhl-10.1177_08903344251318285 for Comparison Between Antenatal and Postnatal Colostrum From Women With and Without Type 1 Diabetes by Alexandra Goldberg, Hans Pettersson, Cecilia Ekéus, Carina Ursing, Eva Wiberg-Itzel and Joanna Tingström in Journal of Human Lactation

sj-docx-3-jhl-10.1177_08903344251318285 – Supplemental material for Comparison Between Antenatal and Postnatal Colostrum From Women With and Without Type 1 Diabetes Supplemental material, sj-docx-3-jhl-10.1177_08903344251318285 for Comparison Between Antenatal and Postnatal Colostrum From Women With and Without Type 1 Diabetes by Alexandra Goldberg, Hans Pettersson, Cecilia Ekéus, Carina Ursing, Eva Wiberg-Itzel and Joanna Tingström in Journal of Human Lactation

sj-docx-4-jhl-10.1177_08903344251318285 – Supplemental material for Comparison Between Antenatal and Postnatal Colostrum From Women With and Without Type 1 Diabetes Supplemental material, sj-docx-4-jhl-10.1177_08903344251318285 for Comparison Between Antenatal and Postnatal Colostrum From Women With and Without Type 1 Diabetes by Alexandra Goldberg, Hans Pettersson, Cecilia Ekéus, Carina Ursing, Eva Wiberg-Itzel and Joanna Tingström in Journal of Human Lactation

sj-docx-5-jhl-10.1177_08903344251318285 – Supplemental material for Comparison Between Antenatal and Postnatal Colostrum From Women With and Without Type 1 Diabetes Supplemental material, sj-docx-5-jhl-10.1177_08903344251318285 for Comparison Between Antenatal and Postnatal Colostrum From Women With and Without Type 1 Diabetes by Alexandra Goldberg, Hans Pettersson, Cecilia Ekéus, Carina Ursing, Eva Wiberg-Itzel and Joanna Tingström in Journal of Human Lactation
