# Supplementary material for: Comparison Between Antenatal and Postnatal Colostrum From Women With and Without Type 1 Diabetes
Source: J Hum Lact. 2025 Mar 12;41(2):254–62. doi: 10.1177/08903344251318285 (PMC11992632; doi:10.1177/08903344251318285)
Supplement: sj-docx-1-jhl-10.1177_08903344251318285 – Supplemental material for Comparison Between Antenatal and Postnatal Colostrum From Women With and Without Type 1 Diabetes [file sj-docx-1-jhl-10.1177_08903344251318285.docx]

**Supplementary Material**

*Number of Missing Colostrum Samples.*

Timepoint T1D Without T1D

GW 36 1 1

GW 37 1^a^

GW 38 3^a^

GW 39 5^a^ 1^a^

GW 40 10^a^ 6^a^

Day 1 2

Day 2 2 3

Day 3 1 1

Day 4 1 1

Day 5 1

*Note.* T1D = type 1 diabetes. GW = gestational week. ^a^Due to birth.
