# Supplementary material for: Comparison Between Antenatal and Postnatal Colostrum From Women With and Without Type 1 Diabetes
Source: J Hum Lact. 2025 Mar 12;41(2):254–62. doi: 10.1177/08903344251318285 (PMC11992632; doi:10.1177/08903344251318285)
Supplement: sj-docx-2-jhl-10.1177_08903344251318285 – Supplemental material for Comparison Between Antenatal and Postnatal Colostrum From Women With and Without Type 1 Diabetes [file sj-docx-2-jhl-10.1177_08903344251318285.docx]

**Table 1b Supplemental**

*Outcome Carbohydrates g/100ml. Estimated Fixed Effects From Mixed Model and Corresponding 95% Confidence Intervals and p-values for Comparisons With Reference Level or Zero for the Intercept.*

| Parameter | Estimate | 95% CI | *p* |
| --- | --- | --- | --- |
| Intercept | 7.95 | [7.59, 8.32] | <0.001 |
| Without T1D | -0.07 | [-0.48, 0.35] | 0.75 |
| T1D | Reference |  |  |
| GW 36 | -1.86 | [-2.27, -1.46] | <0.001 |
| GW 37 | -1.68 | [[-2.07, -1.28] | <0.001 |
| GW38 | -1.96 | [-2.35, -1.56] | <0.001 |
| GW 39 | -2.06 | [-2.46, -1.67] | <0.001 |
| GW 40 | -2.07 | [-2.55, -1.6] | <0.001 |
| Day 1 | -1.75 | [-2.1, -1.41] | <0.001 |
| Day 2 | -0.73 | [-1.06, -0.4] | <0.001 |
| Day 3 | -0.32 | [-0.6, -0.05] | 0.02 |
| Day 4 | -0.13 | [-0.34, 0.08] | 0.22 |
| Day 5 | Reference |  |  |

*Note.* T1D = Type 1 Diabetes. GW = Gestational Weeks. In GW 40 there are only samples from participants without T1D. Example of interpretation: women with T1D at day 5 on average have 7.95g/100ml carbohydrates (intercept). Women without T1D at day 5 have 0.07g/100ml lower fat compared with women with T1D. *P*-value <0.05 is considered statistically significant.
