# Supplementary material for: Comparison Between Antenatal and Postnatal Colostrum From Women With and Without Type 1 Diabetes
Source: J Hum Lact. 2025 Mar 12;41(2):254–62. doi: 10.1177/08903344251318285 (PMC11992632; doi:10.1177/08903344251318285)
Supplement: sj-docx-3-jhl-10.1177_08903344251318285 – Supplemental material for Comparison Between Antenatal and Postnatal Colostrum From Women With and Without Type 1 Diabetes [file sj-docx-3-jhl-10.1177_08903344251318285.docx]

**Table 1c Supplemental**

*Outcome Kcal/100ml. Estimated Fixed Effects From Mixed Model and Corresponding 95% Confidence Intervals and p-values for Comparisons With Reference Level or Zero for the Intercept.*

| Parameter | Estimate | 95% CI | *p* |
| --- | --- | --- | --- |
| Intercept | 73.32 | [63.57, 83.07] | <0.001 |
| Without T1D | 2.65 | [-8.77, 14.06] | 0.63 |
| T1D | Reference |  |  |
| GW 36 | 10.64 | [0.01, 21.27] | 0.05 |
| GW 37 | -4.8 | [-15.18, 5.57] | 0.36 |
| GW38 | -11.23 | [-21.51, -0.94] | 0.03 |
| GW 39 | -13.17 | [-23.44, -2.91] | 0.01 |
| GW 40 | -9.01 | [-21.22, 3.2] | 0.15 |
| Day 1 | -7.85 | [-16.86, 1.17] | 0.09 |
| Day 2 | -5.87 | [-14.32, 2.59] | 0.17 |
| Day 3 | -7.36 | [14.54, -0.17] | 0.04 |
| Day 4 | 0.16 | [-5.22, 5.54] | 0.95 |
| Day 5 | Reference |  |  |

*Note.* Kcal = Kilocalorie. T1D = Type 1 Diabetes. GW = Gestational Weeks. In GW 40 there are only samples from participants without T1D. Example of interpretation: women with T1D at day 5 on average have 73.32 kcal/100ml (intercept). Women without T1D at day 5 have 2.65 kcal/100ml higher kcal compared with women with T1D. *P*-value <0.05 is considered statistically significant.
