# Supplementary material for: Comparison Between Antenatal and Postnatal Colostrum From Women With and Without Type 1 Diabetes
Source: J Hum Lact. 2025 Mar 12;41(2):254–62. doi: 10.1177/08903344251318285 (PMC11992632; doi:10.1177/08903344251318285)
Supplement: sj-docx-4-jhl-10.1177_08903344251318285 – Supplemental material for Comparison Between Antenatal and Postnatal Colostrum From Women With and Without Type 1 Diabetes [file sj-docx-4-jhl-10.1177_08903344251318285.docx]

**Table 1d Supplemental**

*Outcome Protein g/100ml. Estimated Fixed Effects From* *Mixed Model and Corresponding 95% Confidence Intervals and p-values for Comparisons With Reference Level or Zero for the Intercept.*

| Parameter | Estimate | 95% CI | *p* |
| --- | --- | --- | --- |
| Intercept | 1.58 | [1.01, 2.16] | 0.00 |
| Group |  |  |  |
| Without T1D | -0.11 | [-0.94, 0.73] | 0.80 |
| T1D | Reference |  |  |
| Time |  |  |  |
| GW 36 | 4.76 | [3.95, 5.58] | 0.00 |
| GW 37 | 3.02 | [2.21, 3.83] | 0.00 |
| GW38 | 2.26 | [1.42, 3.10] | 0.00 |
| GW 39 | 2.47 | [1.57, 3.36] | 0.00 |
| GW 40 | 3.57 | [2.68, 4.46] | 0.00 |
| Day 1 | 2.61 | [1.84, 3.38] | 0.00 |
| Day 2 | 0.81 | [0.05, 1.57] | 0.04 |
| Day 3 | 0.24 | [-0.39, 0.87] | 0.45 |
| Day 4 | 0.08 | [-0.41, 0.57] | 0.75 |
| Day 5 | Reference |  |  |
| Interaction Time*Group |  |  |  |
| GW 36, Without T1D | -0.80 | [-1.97, 0.36] | 0.17 |
| GW 37, Without T1D | 0.68 | [-0.47, 1.83] | 0.24 |
| GW38, Without T1D | 1.14 | [-0.02, 2.31] | 0.05 |
| GW 39, Without T1D | 0.61 | [-0.59, 1.81] | 0.32 |
| GW 40, Without T1D^a^ | Reference |  |  |
| Day 1, Without T1D | 0.00 | [-1.08, 1.07] | 1.00 |
| Day 2, Without T1D | 0.07 | [-0.97, 1.11] | 0.90 |
| Day 3, Without T1D | 0.01 | [-0.89, 0.91] | 0.99 |
| Day 4, Without T1D | -0.01 | [-0.71, 0.69] | 0.97 |
| Day 5, T1D | Reference |  |  |

*Note.* T1D = Type 1 Diabetes. GW = Gestational Weeks. In GW 40 there are only samples from participants without T1D. Example of interpretation: women with T1D at day 5 on average have 1.58g/100ml protein (intercept). Women without T1D at day 5 have 0.11g/100ml lower protein compared with women with T1D. Due to the interaction, the difference in protein between the groups differs depending on time. For example, women without T1D at GW 36 had lower protein (-0.91 = -0.11 - 0.80) compared with women with T1D.
