# Supplementary material for: Comparison Between Antenatal and Postnatal Colostrum From Women With and Without Type 1 Diabetes
Source: J Hum Lact. 2025 Mar 12;41(2):254–62. doi: 10.1177/08903344251318285 (PMC11992632; doi:10.1177/08903344251318285)
Supplement: sj-docx-5-jhl-10.1177_08903344251318285 – Supplemental material for Comparison Between Antenatal and Postnatal Colostrum From Women With and Without Type 1 Diabetes [file sj-docx-5-jhl-10.1177_08903344251318285.docx]

**Table 1a Supplemental**

*Outcome Fat g/100ml. Estimated Fixed Effects From Mixed Model and Corresponding 95% Confidence Intervals and p-values for Comparisons With Reference Level or Zero for the Intercept.*

| Parameter | Estimate | 95% CI | *p* |
| --- | --- | --- | --- |
| Intercept | 3.54 | [2.52, 4.56] | 0.00 |
| Without T1D | 0.40 | [-0.83, 1.64] | 0.50 |
| T1D | Reference |  |  |
| GW 36 | -0.66 | [-1.71, -0.39] | 0.21 |
| GW 37 | -1.84 | [-2.86, -0.82] | 0.00 |
| GW38 | -2.12 | [-3.12, -1.12] | 0.00 |
| GW 39 | -2.21 | [-3.21, -1.22] | 0.00 |
| GW 40 | -2.17 | [-3.33, -1.02] | 0.00 |
| Day 1 | -1.63 | [-2.50, -0.77] | 0.00 |
| Day 2 | -0.82 | [-1.62, -0.02] | 0.05 |
| Day 3 | -0.81 | [-1.48, -0.13] | 0.02 |
| Day 4 | 0.02 | [-0.48, 0.52] | 0.94 |
| Day 5 | Reference |  |  |

*Note*. T1D = Type 1 Diabetes. GW = Gestational Weeks. In GW 40 there are only samples from participants without T1D. Example of interpretation: women with T1D at day 5 on average have 3.54g/100ml fat (intercept). Women without T1D at day 5 have 0.4g/100ml higher fat compared with women with T1D. *P*-value <0.05 is considered statistically significant.
